# Cannabinoid type-2 receptors: An emerging target for regulating schizophrenia-relevant brain circuits

**DOI:** 10.3389/fnins.2022.925792

**Published:** 2022-08-11

**Authors:** Anthony S. Ferranti, Daniel J. Foster

**Affiliations:** ^1^Department of Pharmacology, Vanderbilt University, Nashville, TN, United States; ^2^Warren Center for Neuroscience Drug Discovery, Vanderbilt University, Nashville, TN, United States; ^3^Department of Pharmacology, Physiology and Neuroscience, University of South Carolina School of Medicine, Columbia, SC, United States

**Keywords:** cannabinoid, cannabinoid type-2 receptor (CB2), schizophrenia, neuropharmacology, psychiatry, dopamine, hippocampus

## Abstract

Although the cannabinoid type-2 receptor (CB2) is highly expressed in the immune system, emerging evidence points to CB2 playing a key role in regulating neuronal function in the central nervous system. Recent anatomical studies, combined with electrophysiological studies, indicate that CB2 receptors are expressed in specific dopaminergic and glutamatergic brain circuits that are hyperactive in schizophrenia patients. The ability of CB2 receptors to inhibit dopaminergic and hippocampal circuits, combined with the anti-inflammatory effects of CB2 receptor activation, make this receptor an intriguing target for treating schizophrenia, a disease where novel interventions that move beyond dopamine receptor antagonists are desperately needed. The development of new CB2-related pharmacological and genetic tools, including the first small molecule positive allosteric modulator of CB2 receptors, has greatly advanced our understanding of this receptor. While more work is needed to further elucidate the translational value of selectively targeting CB2 receptors with respect to schizophrenia, the studies discussed below could suggest that CB2 receptors are anatomically located in schizophrenia-relevant circuits, where the physiological consequence of CB2 receptor activation could correct circuit-based deficits commonly associated with positive and cognitive deficits.

## Introduction

The endocannabinoid system is composed of two main receptor subtypes, cannabinoid type-1 (CB1) and type-2 receptors (CB2) which both belong to the Class A family of G-protein coupled receptors (GPCRs; Pertwee et al., 2010). While CB1 is the most widely expressed GPCR in the brain (Tsou et al., 1998), CB2 has historically been regarded as the “peripheral” cannabinoid receptor due to its prolific expression in immune tissue, such as the spleen and thymus (Munro et al., 1993; Brown et al., 2002; Atwood and Mackie, 2010). However, numerous studies over the past two decades have challenged this dichotomy and functional CB2 receptors have been found in numerous brain regions including the hippocampus, cortex, and dopaminergic circuits (Sagar et al., 2005; Sickle et al., 2005; Ashton et al., 2006; Gong et al., 2006; Onaivi, 2006; Onaivi et al., 2006; Brusco et al., 2008a,b). Unlike most class A GPCRs which respond strongly to a single neurotransmitter, the cannabinoid receptors are activated by several endogenously released endocannabinoids in the brain including 2-Arachidonoylglycerol (2-AG) and anandamide (AEA). In addition to endogenously produced cannabinoids, several phytocannabinoids have been studied including delta-9-tetrahydrocannabinol (delta-9-THC) and cannabidiol (CBD). The endocannabinoid system is complex and involves numerous synthetic pathways and degradation pathways [for a comprehensive review see Lu and Mackie (2021)] providing numerous strategies for modifying endocannabinoid signaling in beneficial ways (Di Marzo et al., 2004; Di Marzo, 2018). The CB2 receptor specifically has emerged as a target for numerous indications including epilepsy (Ji et al., 2021; Shapiro et al., 2021), pain (Beltramo, 2009; Gado et al., 2019), and addiction (Manzanares et al., 2018; Jordan et al., 2020; Navarrete et al., 2021). Here, we will focus on efforts to specifically target the CB2 receptor and the implications for treating schizophrenia.

Members of the endocannabinoid and phytocannabinoid families tend to possess complicated pharmacological profiles and can possess pharmacological activity at both CB1 and CB2 receptors (Pacher et al., 2006; Tham et al., 2019) as well as receptor-independent effects mediated *via* binding to K^+^ channels (Gantz and Bean, 2017; Incontro et al., 2021; Zhang and Bean, 2021). The complex pharmacology of these compounds has made it challenging to elucidate the mechanisms through which delta-9-THC, CBD, and other phytocannabinoids mediate changes in neurotransmission and behavior. Intravenous delta-9-THC administration can induce psychotomimetic effects in healthy volunteers (D’Souza et al., 2004) and cannabis use in adolescents is positively associated with an increased risk for developing psychiatric disorders (Di Forti et al., 2014; Volkow et al., 2016). Collectively these studies suggest that in schizophrenia, the net effect of broad modulation of the cannabinoid system with either delta-9-THC or cannabis use may be detrimental instead of beneficial. Studies in rodents using pharmacological and genetic tools with selectivity for CB1 over CB2 have suggested that the CB1 receptor plays a key role in the psychoactive effects of cannabis use (Monory et al., 2007; Busquets-Garcia et al., 2017), which has encouraged the pursuit of selective CB2-selective ligands as a non-psychoactive target for treating disorders involving the endocannabinoid system. As discussed below, recently developed tools are allowing detailed studies into the role CB2 receptors play in regulating circuits that are hyperactive in schizophrenia including the dopaminergic system, hippocampus, and prefrontal cortex. While more studies are needed to elucidate the utility of targeting CB2 in schizophrenia, the functional expression of these receptors in key brain circuits that are dysregulated in schizophrenia, combined with the anti-inflammatory properties of CB2 receptor activation, make selectively targeting this receptor an intriguing target for treating a disease where novel interventions that move beyond dopamine receptor antagonists are desperately needed.

## Novel tools for cannabinoid type-2 receptor research

The development of pharmacological and molecular biology tools has greatly expanded our understanding of where CB2 receptors are expressed and what functions they play in regulating brain function. The high level of CB2 receptor expression in the immune system compared to the brain led researchers to initially posit that CB2 receptors did not exert a large influence on brain function. However, this hypothesis has since been challenged by numerous studies showing that CB2 receptors are expressed in the brain and can regulate the activity of numerous brain circuits. The brainstem, cerebellum, and cortex were the first brain regions in which CB2 receptor RNA and protein were demonstrated to be expressed in rodents. While the expression of CB2 in the brain is much lower than observed in other tissues such as the spleen, the CB2 receptor expression in the brainstem is sufficient to induce physiological and behavioral efficacy following activation by selective CB2 agonists or endocannabinoids (Van Sickle et al., 2005). Further studies demonstrated CB2 expression in numerous brain regions including the substantia nigra, striatum, hippocampus, and cortex (Gong et al., 2006; Onaivi et al., 2006). However, the finding that CB2 was expressed in the brain was controversial as several studies employing *in situ* hybridization and Northern blotting failed to detect CB2 mRNA expression in the brain (Munro et al., 1993; Galiègue et al., 1995; Schatz et al., 1997; McCoy et al., 1999; Howlett et al., 2002). One possible factor contributing to this controversy is that the levels of CB2 receptor expression may have been low and at a level that is close to the detection threshold for these particular techniques. One key technological advancement in this area has been the development of RNA scope technology that allows multiplexed detection of low expression RNAs with single neuron specificity. Studies employing RNAScope have detected *Cnr2* expression in microglia, as well as hippocampal, midbrain, cortical, and striatal neurons (Zhang et al., 2015, 2021a,b; Stempel et al., 2016; Li and Kim, 2017; Liu et al., 2017). Collectively, these studies indicate that while CB2 receptor expression is highest in immune-related tissues such as the spleen, CB2 receptors are expressed at levels sufficient to regulate central nervous system (CNS) activity in several schizophrenia-related brain circuits as discussed in detail later is this review.

Advancements in developing pharmacological tools have also greatly facilitated studies aimed at elucidating the physiological importance of CB2 receptors to brain function. One of the first strategies employed for developing pharmacological tools was mimicking the tricyclic structure of delta-9-THC, an approach that did not generally produce compounds with high degrees of specificity for CB receptor subtypes, but did yield clinically useful compounds including Nabilone (Pertwee, 2008). Efforts to create analogs of the relatively CB1-selective endocannabinoid AEA resulted in the development of several CB1-selective agonists including AM356 (Thakur et al., 2009) and separate efforts have also identified numerous small molecule antagonists with high selectivity for CB1 including SR141716A and the closely related AM281 (Lan et al., 1999; Pacher et al., 2006). Efforts to create CB2-selective compounds have also been influenced by naturally occurring phytocannabinoids showing subtype selectivity such as β-caryophyllene (BCP) which is a potent and selective CB2 agonist (Gertsch et al., 2008). Given the absence of psychoactive effects observed with CB2 receptor activation, many groups have focused on discovering and patenting CB2-selective compounds (Morales et al., 2016). Many CB2-selective agonists have been developed over the years (Hanus et al., 1999; Murineddu et al., 2013; Han et al., 2014), including JWH-133 which is commonly used in rodent studies. Small molecule CB2 selective antagonists have also been developed such as AM630 (Ross et al., 1999) and SR144528 (Rinaldi-Carmona et al., 1998) which at specific doses has limited permeability across the blood-brain-barrier and thus limited CNS penetration (Bouchard et al., 2012; Lin et al., 2021). The development of CB2 global knockout mice (Buckley, 2008) tissue-selective knockout mice (Stempel et al., 2016; Li and Kim, 2017; Liu et al., 2017) have also provided great tools both for determining the physiological role of CB2 receptors, as well as validating the mechanism of action for CB2-selective compounds. Collectively, these pharmacological and genetic tools have facilitated detailed studies examining how the endocannabinoid system functions under physiological conditions. Moreover, this toolbox is constantly expanding and is aided by recent insights into the structure of the CB2 receptor (Hua et al., 2020; Xing et al., 2020) that have allowed *in silico* approaches to discover novel compounds (Bian and Xie, 2022).

In addition to orthosteric agonists and antagonists that interact with the endocannabinoid binding site, allosteric modulators of CB2 receptors have been discovered that interact with the CB2 receptor at distinct binding sites. These include endogenous peptides such as pepcan-12 which act as positive allosteric modulators (PAMs) at CB2 receptors (Petrucci et al., 2017). While agonists activate receptors upon binding, pure allosteric modulators do not activate receptors directly but change the affinity of agonist binding and/or efficacy of agonist-induced receptor activation. Accordingly, while agonists will activate all the receptors present whenever bound, allosteric modulators can maintain the spatial and temporal patterning of receptor activation to maintain the connection between receptor activation and neurotransmitter release which can lead to different physiological results (Foster and Conn, 2017). It is known that endocannabinoids can be synthesized and released “on-demand” (Kano et al., 2009), and our understanding of the spatial and temporal aspects of endocannabinoid release could greatly expand in the near future thanks to fluorescent sensors that can be used to monitor endocannabinoid levels in awake behaving animals (Dong et al., 2021). Interestingly, EC21a the first small molecule CB2 PAM was recently discovered and has demonstratedantinociceptive efficacy in mouse models (Gado et al., 2019) as well as seizure resistance in a manner that is blocked by co-dosing a CB2 antagonist (Shapiro et al., 2021). While EC21a is an exciting first-in-class small molecule, several efforts are underway to identify other selective PAMs and NAMs (Navarro et al., 2021; Yuan et al., 2022) that have the potential to provide beneficial effects either alone or given with low doses of CB2 agonists (Polini et al., 2020).

As discussed above, it is critical to consider the subtype-selectivity of a given compound, as well as the mechanism of action (agonist vs. PAM), when interpreting pharmacologically induced effects on circuit function and/or behavior. In addition, it is crucial to acknowledge the potential for signal bias at cannabinoid receptors given the multiple signaling pathways that CB2 receptor activation can induce (Wootten et al., 2018). Both CB1 and CB2 are canonically coupled to Gα_i/o_ signaling pathways leading to suppression of cAMP. However, CB2 can also signal through arrestin signaling with compounds often showing a strong bias to one of these pathways, but rarely both (Dhopeshwarkar and Mackie, 2016). A large collaborative effort spanning academia and industry profiled numerous endocannabinoids as well as synthetic CB2 agonists for activity across numerous signaling pathways including cAMP, GIRK, Arrestin, and MAPK pathways (Soethoudt et al., 2017). Given the incredible complexity of CB2 signaling, combined with the off-target activity of compounds, there is a great need to identify which signaling pathways are responsible for different physiological effects. Excitingly, the development of a bitopic CB2-selective compound was recently reported that preferentially inhibits cAMP signaling and maintains anti-inflammatory properties (Gado et al., 2022). While there is much that we still do not understand about what CB2-mediated signaling pathways could be targeted for specific diseases, and more biased compounds are needed to rigorously study the contributions of these signaling pathways, there is great potential moving forward to modulate this system in increasingly precise ways. The growing number of pharmacological tools available to investigate precise mechanisms of CB2 activity will allow researchers to pursue outstanding questions related to the physiological functions of CB2 receptors in regulating neurobiological processes that influence pathological behaviors.

## Cannabinoid type-2 receptor as a target for schizophrenia

Schizophrenia is a complex disease that involves the dysregulation of numerous brain circuits with an onset of symptoms that typically is observed in early adulthood. Symptoms of schizophrenia can include positive symptoms (hallucinations, disordered speech), negative symptoms (anhedonia), as well as cognitive symptoms. The brain areas most associated with schizophrenia include the striatum, the hippocampus, and the prefrontal cortex. In the striatum, hyperactive dopaminergic signaling is highly correlated with positive symptoms and all currently approved schizophrenia medications possess dopamine receptor antagonist activity (Howes and Kapur, 2009). The involvement of glutamatergic pathways has been implicated by the ability of NMDA receptor antagonists to mimic all three disease symptom clusters, and these behavioral effects are mediated *via* disinhibition of pyramidal neurons in the hippocampus and prefrontal cortex leading to hyperactive output from these two brain regions (Moghaddam and Javitt, 2012). While our knowledge of the mechanisms underlying schizophrenia is expanding, the etiology and pathophysiology of this disease are still not completely understood. Evidence for the involvement of the cannabinoid system in schizophrenia is evident by the association with cannabis use and the age of onset for psychosis (Di Forti et al., 2014; Volkow et al., 2016), as well as by the heightened levels of endocannabinoids in cerebral spinal fluid and blood of schizophrenic patients (Giuffrida et al., 2004; Minichino et al., 2019). Changes in the expression of biosynthetic pathways of endocannabinoids have been observed in schizophrenic patients (Volk et al., 2010), as have changes in CB1 receptor expression levels [for review see Volk and Lewis (2016)]. Collectively, these studies suggest that broad-scale activation of the endocannabinoid system may not produce desirable effects with regards to schizophrenia. The phytocannabinoid CBD has been reported to potentially play a role in mitigating the ability of delta-9-THC to induce psychosis (Morgan and Curran, 2008), as well as alleviate psychosis in schizophrenia patients (Leweke et al., 2012; McGuire et al., 2018). However, CBD is pharmacologically complex, and recent reports suggest that the antipsychotic-like and rewarding effects of CBD observed in rodents may be mediated at least in part *via* non-cannabinoid receptors (Galaj et al., 2020; Rodrigues da Silva et al., 2020). Furthermore, some studies have reported no significant effects of CBD in schizophrenia patients (Hallak et al., 2010; Boggs et al., 2018) and relatively few studies have rigorously assessed CBD effects with regards to schizophrenia. While the role of endocannabinoids in the development of schizophrenia, as well as the ability of compounds like CBD to modulate psychosis, are interesting and important topics, here we will focus on CB2-selective modulation as a potential therapeutic for treating schizophrenia after the onset of symptoms.

Some genetic studies have indicated that reduced CB2 receptor expression or activity may indicate an increased risk for developing schizophrenia. In fact, genome-wide association studies (GWAS) from a population-based United Kingdom Biobank sample revealed shared genetic liability between psychotic experiences and schizophrenia and identified a locus in the *CNR2* gene to be significantly associated with distressing psychotic experiences (Legge et al., 2019). Additionally, two single nucleotide polymorphisms (SNPs) in the *CNR2* gene were found to be associated with increased susceptibility to developing schizophrenia (Ishiguro et al., 2010). Interestingly these SNPs were associated with either low levels of gene expression or a loss of function indicating that both would be hypothesized to lead to reduced CB2 activity. This hypothesis that CB2 receptors may play a role in schizophrenia-related behaviors is further supported by rodent studies in which deficits in sensorimotor gating as assessed with prepulse inhibition (PPI) induced by the NMDA receptor antagonist MK-801 can be exacerbated by a CB2-selective antagonist (Ishiguro et al., 2010), and reversed by a CB2 agonist (Khella et al., 2014). This is especially interesting in light of recent reports demonstrating that CB2 receptors are expressed in key brain circuits related to schizophrenia including dopaminergic neurons and the hippocampus. As reviewed below, not only is CB2 expressed in these schizophrenia-relevant brain regions, but activation of CB2 has circuit-level effects that would be expected to normalize hyperactive dopaminergic as well as hippocampal and cortical signaling which would further support the potential of CB2-selective compounds as a novel therapeutic strategy for treating schizophrenia.

## Cannabinoid type-2 receptor mediated modulation of dopaminergic signaling

**FIGURE 1:**
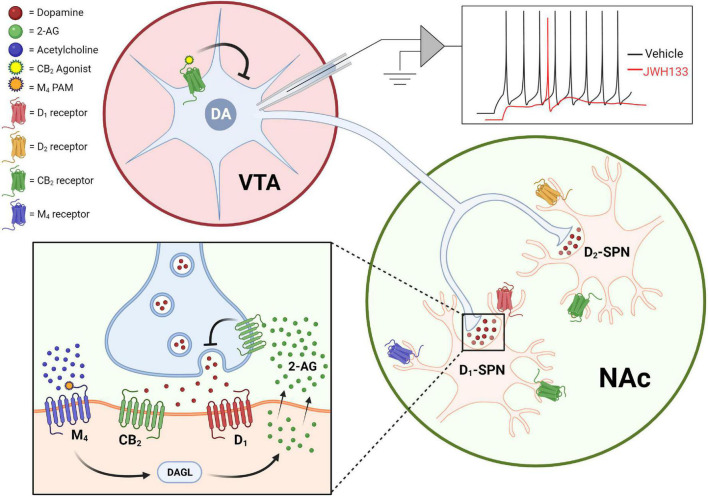
CB2 receptors reduce dopaminergic signaling *via* actions on dopamine neurons somas as well as dopamine neuron terminals. Activation of CB2 receptors expressed in the soma of VTA dopamine neurons suppresses excitability in *ex vivo* cell-attached patch-clamp recordings [top; adapted from Zhang et al. (2014)], while CB2 receptor activation inhibits dopamine release in the striatum and nucleus accumbens (bottom). CB2 receptor-mediated effects on dopamine terminals can be observed both upon direct activation of CB2 receptors with agonists, as well as by mobilization of endocannabinoid and CB2-dependent effects observed after activation of the M4 muscarinic receptor.

Schizophrenia is associated with hyperactive dopaminergic signaling in the striatum with hypoactive dopaminergic activity in the cortex. The hyperactive dopaminergic signaling in the striatum is associated with the positive symptoms of schizophrenia and currently approved antipsychotics are thought to mediate their effects in large part through antagonizing D2 dopamine receptors in this brain region. Drug discovery efforts to find novel treatments for schizophrenia have sought to determine novel mechanisms through which dopamine signaling can be attenuated with the hopes of achieving antipsychotic efficacy with fewer side effects than currently available drugs. The first anatomical evidence that CB2 receptors were expressed in dopamine neurons was initially examined using quantitative RT-PCR and RNAscope to assess mRNA levels and immunohistochemical studies to look at protein expression. These studies found that CB2 mRNA and protein could be detected in the ventral tegmental area (VTA) of wild type rodents (Zhang et al., 2014, 2017), although with important species differences including a higher incidence of neuronal CB2 mRNA expression in mice relative to rats (Zhang et al., 2015). CB2 mRNA levels are detectable in WT and CB1^–/–^ mice, but absent in CB2^–/–^ mice (Zhang et al., 2014) indicating that probes are CB2-specific. Furthermore, the activation of CB2 receptors using the CB2-selective agonist JWH133 induces a hyperpolarization of resting potentials, augmentation of after-hyperpolarization amplitudes, and reduction in the firing rate of dissociated VTA neurons collected from mice or rats (Zhang et al., 2014, 2017). Importantly, these physiological effects of JWH133 in the VTA can be blocked by the CB2-selective antagonist AM630 indicating they are CB2-mediated. Furthermore, dosing of mice with JWH133 reduces VTA neuron firing rates *in vivo*, indicating that CB2 expressed on dopamine neurons is sufficient to induce physiological changes in the intact brain (Zhang et al., 2014). Collectively, these studies provide solid evidence that CB2 is functionally expressed in dopamine neurons where the net effect of CB2 receptor activation on these neurons is a reduction in excitability (Figure 1).

In addition to being expressed on the soma of dopamine neurons, CB2 receptors can also be detected in the brain areas that dopamine neurons most densely innervate including expression on both D1-expressing and D2-expressing spiny projection neurons (SPNs) in the nucleus accumbens (NAc; Zhang et al., 2021b). Remarkably, direct injection of the CB2 agonist JWH133 into the NAc induces significant reductions in extracellular dopamine in the NAc of WT and CB1^–/–^ mice, but not CB2^–/–^ mice, as measured by *in vivo* microdialysis (Xi et al., 2011). Moreover, CB2 receptor modulation can bi-directionally regulate dopamine signaling as direct site administration of the CB2 antagonist AM630 can induce an increase in extracellular dopamine levels in both mice and rats (Xi et al., 2011; Zhang et al., 2017). In addition to directly targeting the CB2 receptor pharmacologically, there is also evidence that cholinergic signaling in the striatum can regulate dopamine release *via* a CB2-dependent mechanism. The M4 subtype of muscarinic acetylcholine receptors is a well-validated preclinical target for treating schizophrenia and activation of M4 receptors with highly selective PAMs can induce antipsychotic-like activity in numerous behavioral assays (Conn et al., 2009; Foster and Conn, 2017). One key mechanism through which M4 PAMs mediate antipsychotic-like efficacy is a reduction of extracellular dopamine levels in the striatum (Byun et al., 2014). Interestingly M4-mediated effects on striatal dopamine release, as well as M4 PAM mediated antipsychotic-like efficacy, are blocked by the CB2-selective antagonist AM630 indicating that CB2 receptors are required for these M4-mediated effects. Furthermore, M4 PAM effects on dopamine release are attenuated by the DAG lipase inhibitor DO34, and are not observed when M4 receptors are deleted from D1-SPNs (Foster et al., 2016), leading to the hypothesis that M4 activation on D1-SPNs induces the release of 2-AG and concurrent CB2 receptor activation that results in a reduction in dopamine release (Figure 1). It is currently unclear what population of CB2 receptors mediates the reduction in dopamine release observed with either M4- or CB2-selective compounds. Further studies will be needed to determine the importance of different CB2 receptor populations including post-synaptic CB2 receptors expressed on D1- and D2-SPNs (Zhang et al., 2021b), as well as presynaptic CB2 receptors expressed on dopamine terminals (López-Ramírez et al., 2020), in mediating CB2 receptor-induced reductions in dopamine transmission in the striatum/NAc.

In addition to physiological evidence that CB2 receptors can regulate dopamine neuron activity, and dopamine release, there is a large amount of evidence that CB2-selective compounds can regulate dopamine-dependent behaviors. The CB2-selective agonist JWH133 dose-dependently reduces cocaine self-administration, cocaine-induced hyperlocomotion, and cocaine-conditioned place preference (Xi et al., 2011; Delis et al., 2017; Lopes et al., 2020), while the CB2-selective antagonist AM630 can block CB2 agonist-mediated reductions in both cocaine-induced hyperlocomotion and self-administration (Xi et al., 2011). Consistent with these pharmacological findings, mice engineered to overexpress CB2 receptors (CB2xP), are resilient to both cocaine motor sensitization and self-administration (Aracil-Fernández et al., 2012), while CB2 KO mice display enhanced cocaine-induced locomotion as well as deficits in sensorimotor gating (Ortega-Alvaro et al., 2011).

Recently the development of tissue-selective CB2 KO mice has provided insights into the importance of this subpopulation of CB2 receptors. Deletion of CB2 from DAT-expressing neurons increases baseline locomotion, rearing, and stereotypic movements, as well as alters the dose-dependent locomotor responses to both cocaine and amphetamine (Liu et al., 2017; Canseco-Alba et al., 2019). In addition, deletion of CB2 receptors from DAT-expressing neurons reduces CPP induced by nicotine or alcohol but does not decrease cocaine- or amphetamine-induced CPP (Canseco-Alba et al., 2019, 2021), indicating that the role of CB2 on dopamine neurons may be substance-dependent. The increased cocaine- and amphetamine-induced hyperlocomotion, combined with the lack of effects in respective CPP observed in the tissue selective CB2 knock out mice, could suggest that CB2 receptors on dopamine neurons do not play a major role in regulating hedonic values. Surprisingly, a selective CB2 inverse agonist (Xie2-64) reduces cocaine self-administration, intracranial self-stimulation, and extracellular dopamine in the NAc of rats and mice (Jordan et al., 2020). However, it is not clear the mechanisms through which Xie2-64 mediates anti-dopaminergic effects, or why deletion of CB2 neurons from dopamine neurons alters the CPP of some substances of abuse but not others. One possible explanation for the actions of Xie2-64, is that it may mediate agonist activity that is either species-dependent or biased in terms of the signal transduction pathways it modulates. However, further studies will be needed to determine the mechanisms through which Xie2-64 mediates its effects. Despite continuing advances utilizing exciting new tools, we still have a poor understanding of the exact mechanisms through which CB2 receptors can mediate effects on dopaminergic signaling. Nonetheless, it is clear that CB2 receptors can modulate dopamine signaling *via* multiple mechanisms including actions at the soma of dopamine neurons as well as in the brain regions where dopamine is released (Figure 1). The majority of results published to date using CB2-selective pharmacological and genetic tools are consistent with CB2 receptor activation resulting in reduced dopamine signaling, an effect that could theoretically be beneficial for hyperdopaminergic diseases such as schizophrenia. However, future studies will be needed to rigorously test the hypothesis that CB2 receptor activation can induce antipsychotic-like efficacy in rodent models and elucidate if this novel mechanism shows promise as a strategy to treat schizophrenia with fewer side effects than currently available therapeutics.

## Cannabinoid type-2 receptor-mediated modulation of hippocampal function

**FIGURE 2:**
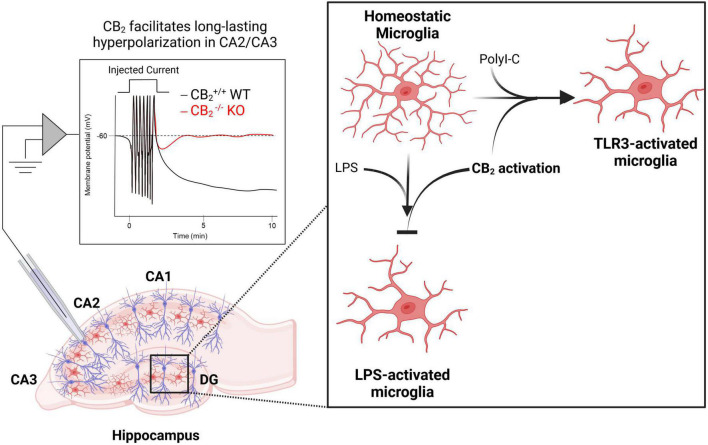
CB2 regulates normal hippocampal functions related to neuronal plasticity, excitability, and inflammation. Activation of CB2 receptors facilitates long-lasting hyperpolarization in CA2/CA3 pyramidal cells [left; adapted from Stempel et al. (2016)]. CB2 receptors also play key roles in regulating inflammation as activation of CB2 receptors both prevents lipopolysaccharide (LPS)-induced microglia activation and is required for toll-like receptor (TLR)-induced microglia activation (right).

Hippocampal hyperactivity is a core feature of schizophrenia and abnormal hippocampal activity is thought to underlie some of the cognitive deficits observed in the disease (Lieberman et al., 2018). Compounds that reduce hippocampal activity therefore could mediate beneficial effects as has been observed with metabotropic glutamate receptors which can regulate cognition *via* changes in hippocampal metaplasticity (Stansley and Conn, 2018; Dogra et al., 2021). Early evidence indicating that CB2 receptors are expressed in neurons came from the identification of CB2 immunoreactivity on apical dendrites of pyramidal neurons in the rat hippocampus (Brusco et al., 2008a,b). CB2 expression was later revealed in the mouse hippocampus among a subset of excitatory and inhibitory neurons using RNAscope *in situ* hybridization (Li and Kim, 2015), and in primate hippocampal neurons (Lanciego et al., 2011). These findings led researchers to investigate the functional significance of CB2 in regulating hippocampal network function. While acute treatment of CA1 pyramidal neurons with JWH133 had no effect on excitatory transmission, chronic activation of CB2 led to an increase in the frequency of miniature excitatory postsynaptic currents (mEPSCs) in CA1 pyramidal neurons, and an increase in the slope of field excitatory postsynaptic potentials in the CA1 region of the hippocampus (Kim and Li, 2015). Additionally, CB2^–/–^ mice display deficits in dendritic spine density, excitatory synaptic transmission, and long-term potentiation (Li and Kim, 2016b). In the CA3 region, action potential trains can drive an endocannabinoid-dependent sustained hyperpolarization that was blocked by the CB2-selective inverse agonist SR144528 and could be mimicked or occluded by a CB2 agonist. Consistent with the pharmacological studies, the long-lasting hyperpolarization was absent in CB2^–/–^ mice (Figure 2), but still observed in CB1^–/–^ mice. Interestingly, this effect appears to require neuronal CB2 receptors as the activity-induced sustained hyperpolarization was absent in mice where CB2 was selectively deleted from neurons (Stempel et al., 2016). Overall, converging evidence points to an inhibitory role for CB2 receptors that causes a hyperpolarization of pyramidal neurons in the hippocampus, and this mechanism could serve as a target to exert neuroprotective and pro-cognitive effects in disorders characterized by excessive neurotransmission and hippocampal-dependent cognitive deficits such as schizophrenia.

The ability of NMDA receptor antagonists to create a schizophrenia-like state is thought to be mediated in large part through disinhibition of hippocampal and cortical circuits (Moghaddam and Javitt, 2012). The capacity of CB2 receptors to hyperpolarize pyramidal neurons, therefore, has the potential to normalize the hyperactivity of pyramidal neurons associated with schizophrenia. Consistent with this hypothesis, CB2 agonists have been shown to reverse NMDA receptor antagonist-induced deficits in somatosensory gating (Khella et al., 2014), a pre-clinical model predictive of antipsychotic-like efficacy. In addition, studies employing CB2^–/–^ mice, as well as CB2-selective pharmacological tools, have revealed that CB2 receptors play a key role in regulating hippocampal-dependent cognitive performance. CB2^–/–^ mice display cognitive deficits as assessed with the step-down inhibitory avoidance paradigm (SDIA) – an assay used to evaluate both short-term (1 h) and long-term (24 h) memory consolidation (Ortega-Alvaro et al., 2011). Furthermore, the CB2 agonist JWH133 dose-dependently enhances both short- and long-term memory consolidation, whereas AM630 dose-dependently diminished SDIA performance in WT mice (García-Gutiérrez et al., 2013). Both male and female CB2^–/–^ mice also show impairments in social memory, however, female CB2^–/–^ mice have significantly higher levels of synapsin-I immunoreactivity in the hippocampus (Komorowska-Müller et al., 2021b), highlighting a need to understand sex-specific differences in CB2 receptor function, an area that is currently under-studied. Additionally, in a model of postoperative cognitive dysfunction (POCD), CB2^–/–^ mice are more vulnerable to isoflurane (Iso)-induced spatial learning and memory impairments compared to WT mice (Li et al., 2021), again suggesting a key role for CB2 receptors in regulating hippocampal-dependent behaviors. While more studies are needed to elucidate the utility of targeting CB2 receptors in treating schizophrenia, these studies collectively indicate that targeting CB2 receptors may represent a novel mechanism to achieve both antipsychotic and pro-cognitive efficacy.

## Cannabinoid type-2 receptors expressed on microglia regulate neuroinflammation

In addition to alterations in dopamine and hippocampal circuits discussed above, inflammation is also thought to play a major role in the disease course of schizophrenia (Kohler-Forsberg et al., 2020). This includes changes in numerous cytokine levels in the blood and cerebrospinal fluid of schizophrenia patients, as well as in the increased comorbidity of schizophrenia with auto-immune disorders (Muller, 2018). Given the heightened levels of inflammation in schizophrenia patients, compounds that can regulate inflammation may be able to provide symptomatic treatment or protect again disease onset or maintenance. CB2 receptors have been demonstrated to play key roles in regulating immune cell function, particularly *via* modulation of microglia (Komorowska-Müller and Schmöle, 2020; Duffy et al., 2021; de Oliveira et al., 2022; Reusch et al., 2022), and the use of CB2 knock out mice have demonstrated that CB2 receptors play key roles in regulating the state of microglia following exposure to different inflammatory responses (Figure 2). CB2^–/–^ mice have altered microglial activity and morphology as well as age-dependent deficits in social memory (García-Gutiérrez et al., 2013; Li and Kim, 2016a; Komorowska-Müller et al., 2021b). Interestingly, old CB2^–/–^ mice (12 months or older) show slightly improved social memory compared to WT controls that displayed an age-related cognitive decline (Komorowska-Müller et al., 2021a), suggesting that CB2 receptors may have age-dependent effects on microglia activity. Genetic tools allowing manipulation of CB2 receptors expressed on microglia have demonstrated that selective overexpression of CB2 receptors on microglia in the CA1 region of the hippocampus enhanced contextual fear memory in a fear conditioning paradigm, while disruption of CB2 receptors in this same population lead to a deficit (Li and Kim, 2017). While more experiments are necessary to piece apart how neuronal vs. microglial expression of CB2 alters distinct behaviors, investigating cell-type-specific manipulations in CB2 expression will allow researchers to better understand the precise mechanisms of how systemically administered CB2-selective compounds mediate behavioral efficacy with relevance to numerous disease phenotypes.

The benefits of CB2 receptor-induced regulation of inflammation could be useful for numerous diseases in addition to schizophrenia. Bilateral injection of amyloid-β into the CA1 area of the rat hippocampus is sufficient to induce deficits in synaptic plasticity, cognition, and memory that are reversed by the CB2 receptor agonist MDA7 (Wu et al., 2013), suggesting CB2 may be a promising target in Alzheimer’s disease. In addition, CB2 appears to play important roles in microglial activation, inflammation, and cognitive disruptions observed following post-operative stress. The expression of CB2 receptors in the hippocampus is upregulated in mice following surgery, and post-operative administration of the CB2 agonist JWH133 can both decrease the expression of pro-inflammatory cytokines and alleviate surgery-induced hippocampal-dependent memory loss (Sun et al., 2017; Li et al., 2021). The ability of CB2 receptors to regulate inflammatory processes could have utility in treating numerous diseases including Alzheimer’s disease (Wu et al., 2013), postoperative recovery (Sun et al., 2017), and neuropathic pain (Cabanero et al., 2020). While more studies are needed to determine if CB2 receptors can influence schizophrenia-related inflammation, the studies above suggest that CB2 receptor activation could potentially have important effects through modulation of microglia function, providing an additional mechanism of action through which CB2 receptors could alter schizophrenia-related pathological changes.

## Concluding remarks

In summary, the CB2 receptor represents an interesting target that can robustly regulate brain circuitry known to be hyperactive in schizophrenia. On dopaminergic neurons, CB2 receptor activation can reduce dopaminergic signaling through multiple mechanisms both at the soma as well as dopamine terminals (Figure 1). In the hippocampus, CB2 receptor activation can lead to a prolonged hyperpolarization of pyramidal neurons, changes in pyramidal neuron morphology, as well as regulate microglial activity to reduce inflammation (Figure 2). In addition to these mechanisms discussed in detail above, the prefrontal cortex is another circuit that is hyperactive in schizophrenia. Excitingly, CB2 receptor expression has been detected in rodent cortical tissue (Miranzadeh Mahabadi et al., 2021; Rivas-Santisteban et al., 2021), as well as in the dorsolateral prefrontal cortex of humans (García-Gutiérrez et al., 2018), with CB2 activation leading to a reduction of pyramidal neuron excitability in the medial prefrontal cortex of rodents (den Boon et al., 2012, 2014). Collectively these findings suggest that CB2 is ideally situated to reduce hyperactive neurotransmission associated with schizophrenia in numerous brain regions. Interestingly, while CB2 was initially regarded as a peripheral receptor, the regulation of dopaminergic and hippocampal-dependent behaviors has been demonstrated to be regulated by neuronal CB2 receptors (Stempel et al., 2016; Liu et al., 2017; Canseco-Alba et al., 2019). However, in some instances the effects of CB2 receptor activation appear to be due to a mix of neuronal and microglial CB2 activation (Li and Kim, 2017), suggesting that multiple CB2 receptors may be able to play a role in regulating brain function. The development of novel pharmacological and genetic tools has greatly enhanced our understanding of how activation of CB2 receptors can regulate brain activity. However, the ability of CB2 receptors to regulate numerous signaling pathways (Soethoudt et al., 2017), can make interpretation of pharmacological results challenging. Advancements in developing CB2-selective pharmacological tools have provided excellent tools for determining the consequences of CB2 receptor activation. Furthermore, the development of genetic tools including tissue-selective CB^–/–^ mice has helped to validate the mechanism of action for these compounds as well as identify the receptor subpopulations that mediate these effects. In the future, the development of CB2-selective compounds showing signal bias could provide even greater insight into the signal transduction pathways through which CB2 receptors mediate their effects on brain function and behavior.

While the studies discussed above indicate that CB2 receptors can regulate schizophrenia-related circuits, there is a relative paucity of evidence that targeting CB2 can mediate antipsychotic-like efficacy. In fact, broad activation of the cannabinoid system seems to be associated with more negative outcomes than positive outcomes (Di Forti et al., 2014; Volkow et al., 2016). However, regulation of CB2 receptors with subtype-selective agonists and antagonists has been demonstrated to bi-directionally modulate preclinical assays predictive of antipsychotic-like efficacy (Ishiguro et al., 2010; Khella et al., 2014). Collectively, the studies reviewed here suggest that CB2 receptors are expressed in a unique pattern in the brain where they can downregulate numerous circuits and inflammatory processes that are hyperactive in schizophrenia patients. In the future, it will be important to expand upon these studies and more rigorously test the hypothesis that selective modulation of CB2 receptors can have efficacy in numerous pre-clinical models predictive of antipsychotic-like efficacy.

## Author contributions

AF designed the figures. Both authors reviewed the literature, wrote and edited the manuscript, contributed to the article, and approved the submitted version.
